# An Empirical Study on the Dairy Product Consumers’ Intention to Adopt the Food Traceability’s Technology: Push-Pull-Mooring Model Integrated by D&M ISS Model and TPB With ITM

**DOI:** 10.3389/fpsyg.2020.612889

**Published:** 2021-01-15

**Authors:** Xin Lin, Run-Ze Wu

**Affiliations:** ^1^School of Economics and Management, Northeast Electric Power University, Jilin, China; ^2^School of Business, Jiaxing University, Jiaxing, China

**Keywords:** food safety, push-pull-mooring theory (PPM), D&M Information System Successful model (ISS), theory of planned behavior (TPB), initial trust model (ITM)

## Abstract

Against the backdrop of frequent food safety problems, the importance of establishing food traceability systems has become increasingly important and urgent to address the contradiction between consumer information on safe food choices and the proliferation of problematic foods. The purpose of this study is to empirically study the influencing factors of Chinese consumers on the food traceability system in the food safety field (hereinafter referred to as FTS). In this study, multiple models—push factor (information system success model), pull factor (ITM theory), mooring factor (TPB), and switching intention—were integrated into the push-pulling-mooring theory (PPM) to form a conceptual PPM comprehensive model framework to study the switching intentions of two-dimensional code traceability technology for dairy products of Chinese consumers. By collecting the questionnaire survey, 305 valid questionnaires were collected from the consumers of middle- and high-end dairy products in China, and the influencing factors of thrust, pull, and mooring force were identified. The results showed that 10 of the 11 hypotheses were positive, but the impact of perceived risk on user satisfaction was negative. The important value of this study is to conduct a comprehensive empirical analysis of the key factors influencing consumer choice of traceable safe food through an integrated multi-model framework to help identify ways to establish and improve consumer willingness to use QR code traceable system products, to increase consumer confidence in the use of traceable and safe food choices.

## Introduction

In the last 20 years, frequent and recurring food safety problems have plagued people around the world. According to an April 2020 survey by WHO, an estimated 600 million people worldwide have fallen ill as a result of eating contaminated food. Unsafe food containing infectious viruses, damaging bacteria, parasites, or chemicals can cause more than 200 diseases, and approximately 3.3 million people worldwide die each year from diarrhea to cancers (Krishnamurthy, 2020). In low- and middle-income countries, unsafe food costs $110 billion a year in lost productivity and healthcare costs (Hou et al., 2019). Also, some researches have confirmed that similar to SARS-CoV-1 and other eight coronaviruses, the SARS-CoV-2 virus is expected to behave similarly at freezing temperatures, which means it could remain contagious under −20°C for up to 2 years (Rizou et al., 2020). Therefore, perfect food safety measures are critical to controlling viral spread (Uddin et al., 2020).

In fact, consumers around the world are often exposed to different degrees of food unsafety effects. Some notorious food safety news have occurred worldwide, including outbreaks of mad cow disease, African swine fever, the Europe horsemeat scandal, melamine-contaminated milk powder, and other incidents. Food safety events seriously endanger both the people’s health and consumer confidence, as well as negatively influence the income of food producers, the development of the food industry, and even the country’s international reputation (Yu and Qiao, 2016).

All these highlight the necessity of implementing or improving the food traceability system (FTS). However, a serious information asymmetry between buyers and producers makes the consumers unable to make informed choices on their food consumption, resulting in a lack of food safety trust. FTS, as an important measure to guarantee food safety, provides each necessary information from farm to plate and makes up the information asymmetry. Because FTS is closely related to food safety and consumers’ claim for traceability and is an important standard of food safety, it is of vital importance to establish FTS to improve consumers’ confidence in food safety (Yu and Qiao, 2016).

Many researches have indicated that traceability awareness and recommendations are issues of specific products and certain countries, and previous work of different researchers mainly focused on analyzing consumers’ preferences for FTS and its influencing factors, and evaluating the role of global consumers in the quality assurance characteristics of food traceability systems, including the United States, Canada, and Korea (Qiana et al., 2020).

As a typical framework of switching intention study, PPM (push-pull-mooring model) reveals some factors that drive individuals to leave their original living places and attract them to new destinations (Wei et al., 2019). Essentially, PPM identifies that push elements drive people away from the origin point, while pull elements attract the crowd to the destination point (Fang and Tang, 2017). Moreover, mooring elements represent the additional elements that promote or restrict judgment of migration (Jung et al., 2017). PPM offers scientists with a clear three-dimensional model of switching intention (Zhang et al., 2014).

Generally speaking, research on switch intention has been lacking an overall framework for the study of transfer intention. Only some researchers have attempted the overall framework, but none have been able to fully integrate the two perspectives of promotion and inhibition. The consumer switch intention study is still emerging on the IS field. However, the problem that the theoretical basis is not comprehensive is still as common as in other disciplines.

This research revealed three series of influencing factors that influence Chinese consumers’ willingness to switch between different brands of milk under the traditional cold chain and the traceability system, and to empirically analyze the influencing relations among the three types of factors. Therefore, this study integrates ISS model, TPB model, and ITM model into PPM, empirically analyzes the three influencing factors in switching intention, and extends the research on the factors in PPM model to draw the conclusion: ISS model, ITM model, and TPB model to analyze the interaction between different variables and switching intention (Fang and Tang, 2017). The results showed that 10 of the 11 hypotheses are positive, but the effect of perceived risk on customer satisfaction is not significant. The aforementioned empirical analysis results proved that it was beneficial to incorporate these three theoretical models into the PPM model. As far as we know, in the field of food safety traceability, there were few empirical studies using PPM integrated model. This study could fill this knowledge gap.

## Background and Literature Review

Frequent food safety incidents have dramatically increased Chinese consumers’ appetite for food safety concerns. The melamine milk powder scandal in China’s dairy industry has weakened Chinese consumers’ confidence in its domestically produced dairy products. With China’ economic development and the increasing domestic income level, milk and other dairy products have gradually increased as a proportion of consumer food consumption, which suggests that milk has become a necessity for many Chinese households.

As a result, the dairy products’ safety has become an important issue in China. In recent years, Chinese producers have undertaken various technology measures (e.g., FTS of dairy industry) to improve the safety of dairy products and enhance the Chinese milk consumers’ confidence (Yin et al., 2016).

From the economic point of view, information asymmetry is one of the problems that contribute to food safety. Obviously, suppliers often utilized information asymmetry between buyers and themselves to engage in opportunistic behavior, such as fraud. If FTS can gain buyers’ trust, it will reduce information asymmetry. FTS has become an important solution for producers to demonstrate the food quality to buyers (Yin et al., 2016). With Chinese consumers’ attention to food safety, Chinese consumers’ demand for FTS has been increasing in recent years (Yin et al., 2016).

### Food Traceability System (FTS)

From an economic point of view, information asymmetry becomes the reason of food safety issues. Moreover, suppliers often misused information asymmetry between buyers and suppliers to carry out opportunistic behavior, for example, fraud (Yin et al., 2016). Facing information asymmetry, traceability systems enable food to be tracked and traced at all stages of production and is regarded by buyers as a vital mechanism to verify the origin of food. Furthermore, it also helps to provide authenticity guarantee for consumers (Kendall et al., 2019).

Food traceability system is a topic that is receiving increasing attention from researchers around the world (Tarjan et al., 2014). The aim of FTS is to determine the food source, protect the food in transit, and reduce the time and cost associated with food recall. A complete FTS, including harvesting, processing, transportation, storage, distribution, and sales, can track products by updating data that are important to the consumers at each stage, such as the product origin, processing mode, storage conditions, and expiration date (Qiana et al., 2020).

To modernize the technical service system, the tools and technologies adopted are continuously developed to realize the progress of the system. Significant advances in information and communication technology (ICT) have promoted the development of FTS both locally and internationally. Barcodes and radio frequency identification (RFID), as identification technologies, have been integrated into FTS to track food products quickly and precisely. Accordingly, FTS has been effectively applied in many different agricultural food industries, including vegetables, fruits, aquaculture, and beef (Qiana et al., 2020).

Facing the information asymmetry, consumers believe that traceability systems that can track and trace food at all stages of production are an important mechanism for verifying the origin of food and also help to provide consumers with assurance of authenticity. Communication with consumers can be facilitated by the development of multiple information delivery mechanisms, including product information linked to the origin of the product and traceability systems. Information technologies consist of barcodes, QR codes, and online material, and can provide more complete information on where products come from and how far along the supply chain they travel.

Barcodes are a perfect choice for speeding up inventory and billing. Barcode is easy to use and is cheap. The Quick Response (QR) code is a two-dimensional bar code that often appears on traceability labels (Qiana et al., 2020). QR code is one of the most commonly used two-dimensional codes. It can store enough data and has very good readability even on small-sized tags, and it is also very readable in the case of physical damage to part of the code (Tarjan et al., 2014). Technological advances in tracking and traceability may further reassure consumers about the authenticity of products, with blockchain technology in particular being seen as a particularly secure and transparent means of ensuring authenticity.

### Pull-Push-Mooring (PPM)

Pull-push-mooring was originally suggested as a specific theory of human migration (Moon, 1995; Lee et al., 2001), which was designed for the dominant pattern of migration research, describing why individuals migrate from one land ecosystem to another terrestrial area (Bansal et al., 2005). Essentially, as a comprehensive framework, it studies the different aspects of users’ migration intention, including the push factors that promote the users’ departure of existing services, the pull factors that attract users to choose an alternative service, and the mooring factor that hinders or promotes the migration decision. PPM derived from migration theory may be a useful conceptual model for analyzing the switching intention because human migration is not just about moving between geographic locations but also extends to a variety of daily activities (Jung et al., 2017).

From the perspective of PPM framework, some researches have solved the switching behavior of the IS sector, such as mobile services (Calvo-Porral and Lévy-Mangin, 2015), travelers’ switching intention (Lehto et al., 2015), the switching intention of social network sites (SNS) (Chang et al., 2014), the switching behavior of web browsers (Potter and Ye, 2011), the attitude change of cloud medical services (Hwang et al., 2015), and the users’ switching behavior of users’ mobile instant messaging (Sun et al., 2017).

Switching intention comes from customers’ comments after using the product or service. When customers’ evolution results are negative, they tend to show switching behavior. The existing literatures have discussed switching behavior based on marketing disciplines and found out a number of factors affecting switching intentions (Hsieh et al., 2012). Due to the increasing popularity of online service activities, there have been more and more studies on switching behavior in the IT/IS field in recent years (Hsin-Ke et al., 2016). Depending on the application contexts, PPM provides researchers with a clear structure to understand the switching behavior of three dimensions (Keng and Hsin, 2019).

One drawback of PPM is that, although PPM is a perfect model for migration studies (Hsieh et al., 2012), when applied to switching intention for food safety, some key factors related to qualities, TPB, and initial trust may be lost (Fang and Tang, 2017). Therefore, we recommend going beyond the existing PPM framework by referring to the actual characteristics of food safety. To our knowledge, the integrated model between PPM, D&M ISS, TPB, and initial trust has not yet been used to explain user switching intentions in the food safety domain. We believe that an integrated PPM will be a suitable framework for this study.

### Push Factors (D&M ISS Model)

DeLone and McLean (2003) suggested a structure for measuring the success of information systems, which has been widely used after its publication. Ten years later, based on the results of empirical tests and theoretical discussions by the academics, they revised their model to measure the information success technologies (DeLone and McLean, 2003). System quality (SYQ), information quality (IQ), and service quality (SEQ) are the key factors, while the results are usage intention, user satisfaction, and net benefit (Chuang and Fan, 2011). For example, SYQ refers to the quality of information automation system, which is manifested as the complete system function. IQ refers to the quality of system output products, including relevance, user-friendliness, adequacy, and accuracy. SEQ refers to the features of the service system that consumers receive from information delivery units and technical supporters, such as friendliness, credibility, and simplicity. User satisfaction is referred as the degree of value produced by IQ (Lee and Lee, 2008).

As one of the popular IS theories to measure the information system technology’s success degree, the D&M ISS model has been widely recognized and applied in various research areas. More than 260 articles have applied this framework to measure the D&M ISS model (Tahar et al., 2013). Particularly, in the context of information system, D&M ISS is applied to many aspects, such as mobile commerce (Lin et al., 2019), mobile banking, and mobile learning (Im et al., 2011).

Even if many evidences have pointed out that ISS can systemically elucidate and predict the factors that influence the usage intention of information system, still few empirical studies on food safety are empirically reported by integrating ISS into PPM.

Perceived risk is generally defined as uncertainty in the possible negative results of utilizing some application or service. Inadequate or unreliable security technologies will enlarge users’ risk degree, leading to reduced satisfaction in the context of information technology. In this study, perceived risk is termed as potential losses or uncertain negative outcomes in the process of adopting food traceability systems, including perceived risks that are often encountered, such as platform failures, missing keywords, operating systems incompatibility, and poor IQ (Chen, 2012). Besides, perceived risk means the possibility that students suffer losses in the process of mobile learning. Students often encounter perceived risks like privacy issues, platform faults, and lost keywords (Chao, 2019).

Relationship quality (RQ) is generally conceptually represented as a complex or multidimensional construct (Sun, 2010). It is a higher-order construct consisting of several distinct but related facets in the relationship (Chen, 2012).

### Mooring Factors: Theory of Planned Behavior Model (TPB)

Famous “intervention barriers” are associated with factors that facilitate human migration, thereby accelerating it and vice versa. This implies that the relationship between variables and individual environment, psychological factors, values, living standards, and social influences is a complementary factor of PPM’s push–pull effect (Kim et al., 2019).

According to the TPB model proposed by Ajzen (1985), certain motivational factors, including attitude toward a certain behavior, subjective norms (i.e., a person’s perception of prohibitive and descriptive norms in a specific group), and perceived behavioral control (PBC), will promote the intention to take a certain action. As long as there is a suitable opportunity, consumers will translate this behavioral intention into actual behavior. TPB is very useful for predicting people’s food choices and their consumption patterns or food handling practices, and as a tool for analyzing the food-choice behaviors in relation with risk or health-related actions (Chen, 2017).

Theory of planned behavior was used to study influencing elements of usage intention and people’s food habits in many areas, for example, a food hygiene intervention, the usage intentions of genetically modified agriculture, and indicators of public perception on risk perception of food additives (Yin et al., 2018).

### Pull Factors: Initial Trust Model (ITM)

The pull factors studied in this paper are structural assurance (SA), personal propensity to trust (PPT), and firm reputation (FR) (Matzembacher et al., 2018). Initial trust is often formed during the first interaction between consumers and mobile payment providers, and it has been confirmed that initial trust was the most critical element affecting the first purchase decision of first-time consumers because wireless transactions can only be executed after the initial trust was established (Matzembacher et al., 2018). The factors that affected the initial trust were determined. Those elements can be roughly divided into three factors. The first style of factors was associated with features of websites. Consumers would depend on their initial perceptual knowledge of mobile payment platform to form the usage intention. Structural assurances were effective in affecting usage intention. The second style of factors was closely connected with company. Firm reputation was also an important element influencing usage intention because it decreased the risk from potential price information asymmetry and after-sales guarantee after finishing the process of mobile payment deal. The third style of factors were combined with user behavior. Personal tendency to trust revealed a tendency and had an important influence to usage intention.

Initial trust model has been applied in many fields to determine the switching intention of information technology, such as mobile shopping, mobile banking, e-commerce, and m-payment. Therefore, FTS needs to be explored to improve the level of food safety. For example, the instability of existing supply chains in the fruit and vegetable industry calls for the establishment of an adequate system and the reduction of numerous food safety incidents and fraud. In the dairy industry, there is a need to ensure quality standards and subsequent consumer satisfaction as products are sensitive to changes in processing and system management can effectively control these changes (Matzembacher et al., 2018).

## Research Model

Push-pull-mooring model has been empirically tested in different fields and identified as a useful information system research framework, such as switching intention of tourists’ hotel booking (Lehto et al., 2015), wireless network websites’ willingness to switch (Zhu et al., 2014), switch to web browsers’ willingness (Potter and Ye, 2011), and changes in attitudes to cloud health service (Hwang et al., 2015); the aforementioned research conclusions indicated that PPM positively influenced switching intention (Keng and Hsin, 2019).

**FIGURE 1:**
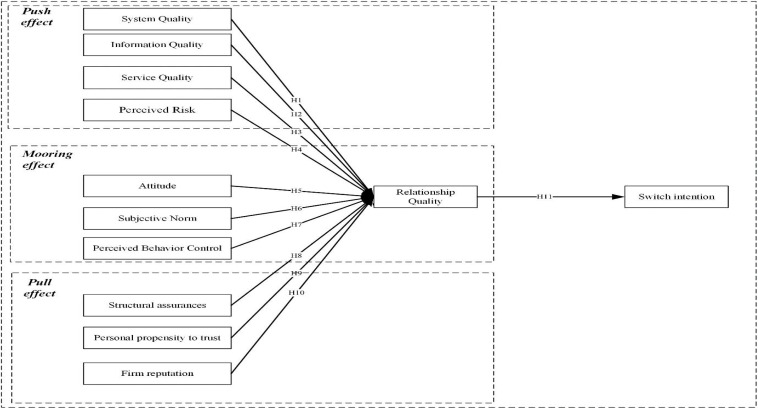
Results of the inner model.

In the Figure 1 of this study, RQ was considered as a higher-level construct with two different but related elements: trust and user satisfaction. In the study of RQ, these two factors were widely referred as the determinate components of RQ (Sun, 2010).

The switching intention of consumers is very important to enterprises. This paper will discuss the factors influencing switching intentions from the perspective of FTS. Generally switching behavior refers to the movement from one dwelling place to another. This movement can also be conceptual, such as replacing an existing service provider with an alternative service provider in the context of a service or using a new product to replace a previously used one. This paper will discuss conceptual switching in the context of both products and services. Switching intention and behavior, as a collective representation known as consumer switching behavior, can have a considerable impact on the performance of dairy companies in the form of retention of old customers and acquisition of new customers. Therefore, a comprehensive understanding of the influencing factors of the consumers’ switching intention can help product and service designs, so as to further expand the benefit scale of dairy companies.

### Push Factors: D&M ISS Model

System quality is referred to as the user’s perceived intensity of the system’s ease of operation, the intensity of connection and learning, and the degree of pleasure in use (Petter and McLean, 2009). Rizal et al. (2018) pointed out that the perceived SYQ describes the individual’s evaluation of the system performance characters based on the user’s experience in using the system. Chung et al. (2014) argued that they focused on the influential factors such as the convenience, system reliability, response time, and flexibility of mobile business access terminals in Korea to identify how SYQ has become a key variable in service loyalty. In the absence of these functions (Sharma and Sharma, 2019), users may doubt the ability of mobile payment service providers to provide quality services, which may increase the difficulty of using their devices.

Therefore, SYQ may affect user satisfaction, and we predicted the following hypothesis:

H1. System quality significantly influences relationship quality.

DeLone and McLean (2003) confirmed the IQ’s characteristics, such as integrity, accuracy, pertinence, and accessibility, which are fundamental factors for use and indicate that IQ is a vital element in determining consumers’ intention to choose some technology that they will use. Hence, IQ should be considered as the core structure affecting user satisfaction and intention.

Rizal et al. (2018) argued that in an environment mediated by a food safety traceability system, customer’s purchasing willingness on some enterprise products and services can be determined by their perceived IQ. Research also suggests that the IQ of dairy products is an important component in building a positive reputation (DeLone and McLean, 2003). Therefore, the hypothesis is suggested as follows:

H2. Information quality significantly influences relationship quality.

Service quality (SQ) refers to the features (e.g., reaction, trustworthiness, brevity, and technological capability) of the service that consumers receive from the information systems divisions and after-sales service. SQ is often termed as how information service fulfills user needs. Only when it comes to the quality of services can the effectiveness of information systems be correctly evaluated. If the quality of service is not involved, the effectiveness of the information system cannot be evaluated correctly. We believe that confirming all quality factors, including service quality, will help consumers to set up and measure the RQ accurately.

Service quality also influences use satisfaction, perceived quality, and service conversion. For example, customers with high service quality are evaluated to show positive intention. Contrarily, customers with low service quality will tend to choose a change agent over a new purchase in the next consumer choice situation (Rizal et al., 2018). Considering the aforementioned situations, the following hypothesis is proposed:

H3. Service quality significantly influences relationship quality.

It is believed that perceived risk (PR) refers to a person’s perception of the ambiguous and adverse results of an action (Glover and Benbasat, 2014). PR plays an important role in decision-making (Rahman et al., 2019). In this study, PR is defined as the combination of uncertainty and the severity of the results involved. According to risk theory, there is an acceptable range of risk; when the risk perception exceeds that, consumers will take solutions to control the level of risk under an acceptable level. The higher the risk perception is, the more likely consumers are to take actions to reduce the risk.

From the perspective of the PPM model, because many studies have shown that push effects often include negative perceptions of service providers, such as service failures, staff problems, and price problems, leading to low satisfaction and trust, this research attempts to explain how FTS has become an important supplement to food safety push factor. Therefore, we suggest the hypothesis as follows:

H4. Perceived risk significantly influences relationship quality.

### Mooring Factors: TPB Model

Theory of planned behavior is in the most significant social psychological theories to predict human activities (Chen, 2017). TPB consists of three components, for example, attitude, subjective norm, and PBC. Taken together, behavioral beliefs produce behavioral attitudes, normative beliefs create subjective norms, and control beliefs lead to perceived behavior control. The combination of all these factors results in a behavioral intention.

Even if TPB has been successfully used to predict switch intention and behavior in a number of fields, it may not gather all the elements of the special behavior, for example, the food choice decision (Chen, 2017). TPB should comprise more components to better predict switch intention and behavior.

Attitude toward a special behavior is an overall evaluation of an individual’s goal behavior (Ajzen, 1985). Many researches have confirmed the influence of attitudes on switch intention (Yin et al., 2018).

H5. Attitude significantly influences relationship quality.

Subjective norms are prerequisites for the implementation of social behavioral intentions, including the application of information technology (Ajzen and Fishbein, 2005). Consumers’ switch intention is also a function of their cognition of the subjective norms toward switching to the alternative.

The role of subjective norm makes consumers naturally change their habitation based on some norm or majority opinion (Ajzen and Fishbein, 2005). Numerous studies have revealed that mandatory and descriptive norms have an impact on behavioral intention. When the public is faced with reporting dilemmas, the more influence a person or organization has over them, the more pressure they feel to participate in food safety reporting, thus enhancing their intention to participate (Yin et al., 2018).

H6. Subjective norm significantly influences social intention.

Perceived behavioral control is defined as the perception of how easy it is for people to perform an act of interest (Ajzen, 1985) or the degree to which an individual thinks the act is controlled by his or her willingness (Price et al., 2015). PBC determines the degree of difficulty for an individual to perform a certain behavior, which can not only directly affect the behavior but also indirectly affect the behavior through intention. Public food safety reporting behavior can be affected by many objective factors (Yin et al., 2018). Given this situation, this hypothesis can be concluded that:

H7. Perceived behavior control significantly influences switch intention.

### Pull Factors

According to the definition of the pull effect in the PPM framework, the pull effect is the positive effect of encouraging potential migrants to travel to a new destination (Keng and Hsin, 2019).

The initial trust model in an individual’s perception of the institutional environment is related to differences in security or procedural safeguards. In previous research, institutional trust is a structural guarantee.

Structural guarantees are related to evaluation and success through institutional devices such as regulations and legal guarantees under specific circumstances. They are composed of trustworthy guarantees, regulations, commitments, and legally binding structures. Especially in cross-border e-commerce transactions, the danger of being late is greater because of the strangeness recognized by customers. In a way to reduce the risk of users being late, structural guarantee becomes an important variable. Therefore, the hypothesis postulates:

H8. Structural assurances significantly influence relationship quality.

The personality-based personal trust tendency is the trust that is formed from the small and can be said to be the general tendency of others. Such personal trust tends to be believed to be generally straight and dependent, and the transaction with the trusted person will have a better result before the trusted person takes the opportunistic action.

Therefore, personal trust tends to have no experience with the trustee and depends on trust expectations. Personal trust tends to have a significant impact on initial trust in the initial trust situation.

Research by Wu and Lee (2017) shows that if a company provides a safe and accurate service, the customer’s trust tendency will have a significant impact on the usage intention of the service. Therefore, the hypothesis postulates:

H9. Personal propensity to trust significantly influences relationship quality.

Company reputation is one of the important factors that affect early credibility. For mobile applications, two main factors affect reputation: the certification mark and brand recognition. Studies by Wu and Lee (2017) have shown that company reputation has a positive impact on initial trust. Therefore, the hypothesis postulates:

H10. Firm reputation influence significantly influences relationship quality.

Relationship quality plays an important role in exploring the connections that exist between customers and the enterprise. Many academics have researched the influence of the three components of RQ on switching intention. For instance, Sanchez-Franco (2009) researched the effects of satisfaction and trust on loyalty of Internet technology providers. They found that satisfaction significantly affects commitment and trust. In addition, Hennig-Thurau and Klee (1997) studied the impact of RQ dimensions, such as customer satisfaction and trust, on customer’s switch intention. Jani and Han (2011) studied the impact of customer satisfaction, trust, and commitment on behavioral intentions, and found that customer satisfaction had a direct impact on intention.

H11. Relationship quality significantly influences switching intention.

## Data Collection and Results

In the preliminary test, this study referred to relevant literature and expanded various variables and related measurement items according to the theme of this study. We invited 50 Chinese consumers who often use the blockchain QR code for dairy product safety trading and completed the prediction test through face-to-face interview with the designed questionnaire. Then the ambiguous measurement items were modified so that the follow-up consumers could fully understand the content of the questionnaire and improve the credibility of the follow-up survey. In the follow-up questionnaire, we recruited 300 consumers (220 Chinese and 80 South Koreans) who had used the blockchain QR code dairy products. Because the South Koreans in northeast China are more concentrated than those in other parts of China, we can get enough samples quickly. In the large supermarkets of northeast China, we randomly intercepted consumers and identified the right people who have used the blockchain QR code to buy safe dairy products. After receiving the questionnaire, appropriate users filled in the questionnaire according to their own intention.

From the beginning of August to the beginning of September in 2020, the survey conducted a 3-week one-to-one interview on the traceability use intention of dairy products in major supermarket chains in northeast China. Participants who are not involved in QR technology are completely excluded. A total of 350 questionnaires were issued and 330 were recovered, with a response rate of 94.28%. After 25 responses were discarded, 305 samples (92.42%) were used for deterministic analysis due to lack of critical data or experience with QR codes. The final data are sufficient to define the sample. Items were provided on a five-point Likert scale (Table 1), ranging from “strongly reject” to “strongly agree.” In the subsequent SEM analysis, IBM SPSS 25.0 was used for evaluation to check the element structure and internal correlation of each part. In this study, Cronbach’s α was used to evaluate the validity of the structure by examining the factor structure and internal correlation of each structure. To test the study hypothesis, we used IBM Amos 24.0 to determine the causal relationship by means of significance values and standard coefficients. The entire sample is used to analyze the integration model before hypothesis testing.

**TABLE 1:** Sample characteristics.

Characteristics	Description	Frequency	Percentage (%)
Group	China	305	100
Gender	Female	222	72.79
	Male	83	27.21
Age	Below 30	205	67.21
	30–40	45	14.75
	40–50	40	18.04
	Above 50	15	3.04
Occupation	Company employee	155	50.52
	Civil servant	85	27.88
	Self-employed person	40	13.11
	Others	25	8.49
Experience for QR	Yes	305	100.0

### Reliability, Validity, and Measurement Model Evaluation

We evaluated the measurement item’s convergence effect on its related structure. First, the standardized load method is used to measure reliability. Moreover, Cronbach’s α and CR scale were used for reliability measurement. Furthermore, AVE is extracted to measure the variance of a variable relative to the variance.

As shown in Table 2, Cronbach’s α is generally better than 0.60, and CR is also higher than 0.80 (Nunnally, 1978), indicating that the optimal validity measurement explains the structure of the scale and the overall consistency level is high. In addition, the aggregate validity is a measure of three-dimensional factors, that is to say, the standardized load represents the relationship between some basic factors and each indicator is 0.7 statistics, except that the overall reliability of the reliability scale is significantly greater than 0.6 (Nunnally, 1978; Hair et al., 1998); each AVE value is greater than 0.6 (Fornell and Larcker, 1981).

**TABLE 2:** Convergent validity and reliability (entire samples).

Construct	Indicators	Standardized loading	Cronbach’s α	Composite reliability	AVE
SYQ	SYQ 1–4	0.750–0.836	0.862	0.8631	0.6122
IQ	IQ 1–4	0.759–0.814	0.871	0.8551	0.5968
SEQ	SEQ 1–4	0.753–0.879	0.872	0.8649	0.6166
PR	PR 1–4	0.690–0.846	0.851	0.8519	0.5910
AT	AT 1–4	0.824–0.854	0.900	0.9004	0.6932
SN	SN 1–4	0.734–0.788	0.855	0.8552	0.5965
PBC	PBC 1–4	0.762–0.847	0.880	0.8819	0.6516
SA	TP 1–4	0.856–0.910	0.933	0.9344	0.7808
PPT	PPT 1–4	0.797–0.852	0.904	0.9037	0.7013
FR	FR 1–4	0.734–0.876	0.877	0.8800	0.6483
RQ	RQ 1–4	0.811–0.866	0.903	0.9039	0.7019
SWI	SWI 1–4	0.849–0.907	0.930	0.9302	0.7691

As shown in Table 3, discriminant validity refers to the difference between the related indexes of the first principle and those of the second principle (Bagozzi et al., 1991). Fornell and Larcker (1981) found that the discriminant validity test must be carried out by evaluating the square root of AVE in each variable in the correlation coefficient of each construct.

**TABLE 3:** Discriminant validity (entire sample).

	SYQ	IQ	SEQ	PR	AT	SN	PBC	SA	PPT	FR	RQ	SWI
SYQ	0.782											
IQ	−0.250	0.773										
SEQ	−0.199	0.166	0.785									
PR	−0.147	−0.169	0.054	0.769								
AT	−0.020	0.259	0.301	0.104	0.833							
SN	−0.045	0.221	0.247	−0.053	0.518	0.772						
PBC	−0.061	−0.126	0.166	−0.034	−0.501	−0.391	0.807					
SA	0.163	0.087	0.082	−0.104	−0.254	−0.244	0.186	0.884				
PPT	0.132	0.128	0.203	−0.108	0.336	0.197	0.252	0.502	0.837			
FR	0.071	0.146	0.231	−0.114	0.271	−0.222	0.172	0.341	0.461	0.805		
RQ	0.134	0.342	0.348	−0.175	−0.524	0.461	0.381	0.420	0.491	0.453	0.877	
SWI	0.055	0.181	0.337	−0.100	0.491	0.360	−0.392	0.495	0.519	0.464	0.745	0.838

Judged by Table 3, for every data, it can be seen that for each data, the square root of the variance between each structure and each AVE is greater than any correlation coefficient between the relationship structure between the structure and another, and the meeting’s good discriminative validity criterion. The correlation between constructs exceeds the diagonal value, which proves that the construct validity of our measurement tool is satisfactory.

The two-process method (Anderson and Gerbing, 1988) was used to evaluate the selected data. First, we examined the convergence and effectiveness of the algorithm. The second step was to evaluate the integration framework between the portfolio models. Third, to test the fitting degree and structure of the measured values, the data of Chinese consumers were applied to test the overall structure. The following model fitting indexes were recommended by Hooper et al. (2008).

The AMOS 24.0 program was used to assess this study’s measurement and structural framework. The χ^2^/d.f. are 1.156 and 1.108, GFIs are 0.850 and 0.845, AGFIs are 0.820 and 0.826, NFIs are 0.894 and 0.894, CFIs are 0.988 and 0.978, IFIs are 0.988 and 0.978, RFIs are 0.882 and 0.853, PGFIs are 0.840 and 0.758, PCFIs are 0.888 and 0.907, PNFIs are 0.803 and 0.796, RMRs are 0.040 and 0.060, and RMSEAs are 0.019 and 0.025. Our results support this relationship of each model.

The 11 hypotheses of this research were examined by SEM. For the contraction fitness index, it exceeds the acceptable minimum fitness value, which is a standard value. The fitting indexes show that the fitting results of the analytical sample and the comprehensive model are satisfactory.

### Hypothesis Verification

**FIGURE 2:**
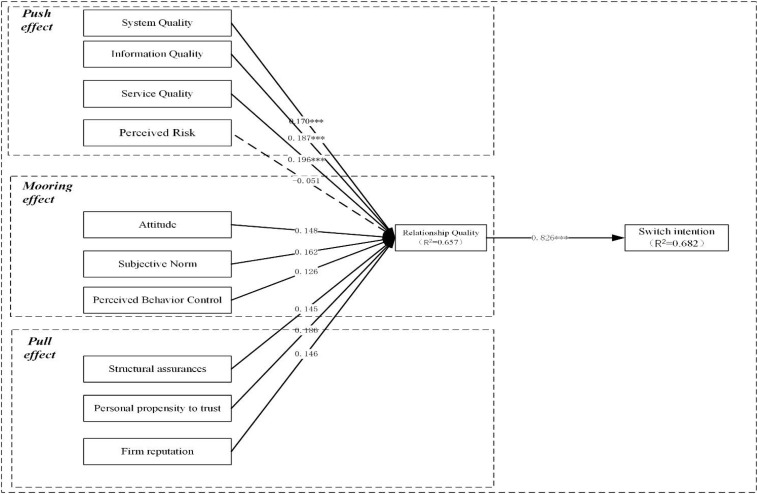
Research model.

After examining measurement suitability and integrated framework’s organization, the structure’s path coefficient was estimated. According to the *p*-value in Figure 2, one path (H4; *p*-value of >0.05) was rejected, and the remaining nine paths were confirmed as positive.

The Chinese consumers’ switch intention that was influenced by SYQ (β = 0.17), IQ (β = 0.187), SEQ (β = 0.196), PR (β = −0.051), AT (β = 0.148), SN (β = 0.162), PBC (β = 0.126), SA (β = 0.145), PPT (β = 0.186), FR (β = 0.146), and RQ (β = 0.826) jointly explained 68.2% intention of switch variance.

The test results and structural model were given, and the model of FTS experience is analyzed. Judged by Table 4, the existence of the comprehensive model is verified. Table 4 verifies the correlation path coefficient, lists the causal path characteristics, and verifies the results of the combined framework. The basic assumptions’ path coefficients of the synthesis model were well measured. According to the respective p values, one path (H4; *p* > 0.05) was not supported, and the remaining paths were significantly lower than 0.05 level.

**TABLE 4:** Results of hypotheses tests.

Hypothesis	Route	Estimate	SE	*T*-value	*P*	Path coefficients
H1	SYQ→RQ	0.183	0.054	3.413	***	0.170
H2	IQ→RQ	0.166	0.044	3.798	***	0.187
H3	SEQ→RQ	0.196	0.050	3.917	***	0.196
H4	PR→RQ	−0.052	0.047	−1.114	0.265	−0.051
H5	AT→RQ	0.138	0.061	2.268	0.023	0.148
H6	SN→RQ	0.152	0.056	2.722	0.006	0.162
H7	PBC→RQ	0.122	0.053	2.3	0.021	0.126
H8	SA→RQ	0.113	0.041	2.794	0.005	0.145
H9	PPT→RQ	0.171	0.055	3.103	0.002	0.186
H10	FR→RQ	0.136	0.049	2.794	0.005	0.146
H11	RQ→SWI	0.979	0.07	13.947	***	0.826

*p < 0.05; **p < 0.01; ***p < 0.001. RQ, relationship quality; SYQ, system quality; IQ, information quality; SEQ, service quality; PR, perceived risk; AT, attitude; SN, subjective norm; PBC, perceived behavior control; SA, structural assurances; PPT, personal propensity to trust; FR, firm reputation; SWI, switch intention.

Judged by the previous analysis results, we can draw the following conclusions: (1) SYQ is a key factor determining RQ and has a significant positive impact on RQ. So H1 is valid. (2) In the concept of a user-friendly interface, navigation convenience, response time, and reliability are the most important variables. There is also a significant positive relationship between IQ and RQ, among which security, integrity, correlation, and privacy protection are the most important variables. Therefore, H2 is valid. (3) Service quality has a significant positive effect on RQ. Therefore, H3 holds. Ability to complete transactions and after-sales service, and meet customer’s demands are most critical to service quality. (4) Perceived risks have no positive influence on RQ. Thus, H4 failed.

Relationship quality plays a critical role in fully mediating variables between SYQ, IQ, SEQ, and switching intention. The SYQ, IQ, and service quality of dairy sellers using the blockchain QR code can enhance customers’ trust in the enterprise’s ability, integrity, and goodwill, and promote dairy consumers to enhance their purchase intention. Thus, H11 is established.

Moreover, our combined TPB model with PPM theory also shows that RQ is a major factor in influencing the switching intention of choosing dairy products. This conclusion is consistent with other researches which have found that RQ is a vital motivational driver in the intention-switching procedure (Glaeser et al., 2000; Hobbs and Goddard, 2015). Thus, H5–7 are established.

In addition, as an additional variable to explain the intention of Chinese dairy consumers, our measurements of structural guarantees, PPT tendency, and firm reputation are statistically significantly positive. Our results confirm that H8–10 are true: initial trust variables in QR code positively affects RQ.

### Mediation Model Verification

The mediating effect was analyzed with Bootstrap and Amos 25.0 software, and the results are shown in Table 5.

**TABLE 5:** Mediating effect analysis.

Independent variable	Mediating variable	Dependent variable	Mediation effect	SE	LLCI	ULCI	*P*-value
SYQ	RQ	SWI	0.14	0.046	0.053	0.23	0.002
IQ	RQ	SWI	0.155	0.046	0.065	0.241	0.002
SEQ	RQ	SWI	0.162	0.046	0.07	0.249	0.001
PR	RQ	SWI	−0.042	0.037	−0.116	0.029	0.269
AT	RQ	SWI	0.122	0.059	0	0.233	0.05
SN	RQ	SWI	0.133	0.052	0.033	0.24	0.014
PBC	RQ	SWI	0.104	0.051	0.006	0.21	0.033
SA	RQ	SWI	0.12	0.045	0.035	0.214	0.016
PPT	RQ	SWI	0.153	0.048	0.06	0.247	0.001
FR	RQ	SWI	0.12	0.046	0.028	0.206	0.009

*p < 0.05; **p < 0.01; ***p < 0.001.

In Table 5, when the independent variable is PR, the mediating effect results is −0.042, and the CI is −0.116 to 0.029, concluding 0. The significance *p* = 0.269 > 0.05, indicating that the independent variable PR cannot influence SWI through RQ.

Through the aforementioned SEM tests, we concluded that PR negatively influenced switch intention because significance *p* = 0.269 > 0.05. This conclusion indicates that FTS providers should increase other quality factors (SYQ, IQ, and SEQ) to influence QR and decrease ITM (SA, PPT, and FR) to stimulate users’ usage intention, to retain the old consumers, which will drive online service providers to generate more competitive features to attract new consumers.

In addition, we have tested the relationship among PPM, ISS, ITM, and dairy consumers’ intention to buy, and the significant direct effect of the causal relationship has been proved. The RQ plays a critical and sufficient mediating role among PPM, ISS, ITM, and dairy consumers’ switching intention.

## Conclusion

### Discussion

With the passage of time, the importance and relevance of FTS become more and more obvious. Our result shows that traceable dairy products benefit Chinese consumers. Chinese consumers influenced by traceability food information are willing to pay the extra price for the traceable information. Chinese consumers’ lack of information on traceable dairy products may be seen as an obstacle to the development of the traceable dairy industry. Therefore, further task is needed to raise consumer awareness of the potential advantages of traceability systems in the dairy product systems.

In this paper, it is necessary to use the PPM model in the migration theory to explain the switching intentions of customers on the dairy products. Second, according to the common characteristics among the first-order dimensions that influence the customer’s switching intentions, this study classified factors into three and validates the usefulness of second-order construct framework to conceptualize the model more concisely. In this study, we focus on the factors that can influence and promote consumers’ switching intention to increase or decrease the use of FTS. Therefore, like Hoffman and Novak (2009), we combined TPB and ITM with ISS and PPM as the prerequisite of the framework. In particular, we identify the significant factors influencing push–pull. This study shows that all the other 10 hypotheses are valid except PR.

### Theoretical Contribution

This study is of great significance to researchers and practitioners. To address the deficiencies in both the theoretical structure and empirical analysis in food safety FTS, three series of factors (i.e., the D&M ISS model, the TDB model, and the ITM theory) are combined with the PPM to form a complete integrated model as a conceptual framework. The conceptual framework enhances the explanatory deficiencies of the three separate models and further clarifies the subjective and objective factors that influence conversion intentions. This study builds a complete multidimensional framework for food safety FTS (shown in Figure 1). It is proposed that SYQ, IQ, SEQ, PR, AT, SN, PBC, SA, PPT, and FR are 10 important determinants to measure the switching intention of FTS. By emphasizing the validity of integrating the three established theories of ISS, TPB, ITM, and PPM, this study provides a holistic approach for future researches using the new FTS technology, namely blockchain two-dimensional code technology.

Our results show that the proposed model has strong explanatory power and is robust in several cases. The integration framework has not only theoretical appeal but also important empirical significance. The following inspirations can be obtained from this study:

#### Push Effects

Some enlightenments are obtained from this study. First of all, our research develops the previous researches. The research results take IQ, SYQ, and SEQ as a whole and provide support for the research on information SYQ. (1) SYQ: research shows that SYQ is the key factor determining trust. Only in all transactions where the system promotes a quality process at all times can high-quality operations be produced. The quality of blockchain QR code technology can enhance customers’ experience of these activities. (2) IQ: it is found that quality of information has a significant impact on the quality of relationships. Previous studies have also confirmed that security, privacy, relevance, and integrity are important for establishing RQ (Liu and Arnett, 2000; Lee and Turban, 2001; Gefen, 2002; Gefen et al., 2003). (3) SEQ: research shows that service quality is also a key factor determining the quality of relationships. If the quality of service provided by milk product operators adopting blockchain two-dimensional code technology can meet the expectations of customers, it can cultivate customers’ trust. Milk product operators adopting blockchain two-dimensional code technology should pay attention to customers’ needs, provide follow-up service support for customers, and present the realization of promises in an efficient way, which will increase customers’ trust belief and ultimately encourage purchase intention.

According to our results, there is an insignificant relationship between perceived risk and RQ. Risk is the probability distribution of the outcome of an event, the gain or loss of which is uncertain. As consumers cannot timely and accurately understand the impact of food on their own health, the uncertainty of food safety risks is relatively high. This information asymmetry challenges the users’ perceived risks conception. Because of the information asymmetry, ordinary users will only count on feelings and experience to make judgments instead of depending on scientific details. This is often at variance with the actual situation of food safety. For example, consumers may exaggerate to treat food safety emergencies like bird flu and mad cow cases, even though the frequency of that is rather low, and the risk is low. However, some serious and high-risk food safety events are overlooked, for example, the long-term consumption of many fried foods that are carcinogenic. As a result, there is a large discrepancy between consumer perceptions and facts.

#### Mooring Effects

First of all, attitude positively affects the switching intention of dairy consumers. Obviously, the dairy products providers should foster positive attitudes toward food safety RQ among dairy consumers and raise their awareness of the importance of reporting food safety RQ. This measure can cultivate dairy consumers’ high sense of social responsibility and enhance their food safety awareness.

Second, PBC also positively affects the switching intention of dairy consumers in choosing safety food. Therefore, the dairy product providers should focus on decreasing difficulty in choosing safety dairy products. The dairy product providers should develop innovative, effective use of the Internet and big data to streamline the food safety reporting process, smooth the multiplicity of reporting channels, and ensure that public food safety reporting is simple, convenient, and feasible.

Third, subject norm is a major determinant whether a consumer participates in food safety report, which leads to the emphasis that participating in food safety report is an individual’s social ideal behavior and moral obligation. This need reinforces their personal perception that it is morally right to participate in food safety reporting.

#### Pull Effects

From the dairy product consumer’s perspective, a perceived benefit of traceability, which can impact all stages of the supply chain, is that it provides additional product-related information and enhances the consumers’ RQ. In addition, these systems can respond quickly in the event of a food safety incident, helping to keep dairy products’ trust. In the event of an incident, traceability information can shift responsibility to the source of the safety issue and avoid reputational damage due to the mistakes of different actors.

The emergence of new technologies in the food system reduces consumers’ understanding and familiarity with food production and separates them from the food producers and processors. It is necessary to provide structural assurances such as “transparent information that marks its quality and safety.”

If a dairy company’s consumer-facing communication is easy and honest, combined with blockchain QR code technology, it will be able to create a positive image for the brand and the company’s products will be perceived as high quality, thus generating a positive reputation for the company’s products. In fact, a brand that includes transparent information about its quality and safety can be perceived as a high-quality and safe food product. When dealing with traceability systems, firm reputation is important to retain the same customer base, and corporate goodwill offers the possibility of gaining a unique position in the marketplace.

Consumer propensity to trust tends to pay more for traceability information on labels, and the characteristics most willing to pay a premium are those of open, serious, outgoing consumers, and those with material and physical needs. Structural assurance, firm reputation, and PPT positively influence RQ, which positively influences switching intentions.

### Managerial Implications

The management significance of this study is shown in the following aspects. First, it is helpful for FTS platform designers to recognize the major influencing factors for maintaining existing users and attracting new learners. Second, the conclusion can systematically and empirically explain the main elements affecting the switch intention, and also help the government to fill the gaps in the FTS’s theory and practice when strengthening food safety. Third, the main factors obtained from the results will help the food industry to accelerate the construction of digital FTS and improve the efficiency of food traceability practice, so as to strengthen the systematic loopholes in food safety and the prevention and control of problematic food by using high-tech teaching methods.

Based on the aforementioned conclusions, we propose the following suggestions. First of all, after the occurrence of food safety incidents, the government and relevant regulatory authorities should promptly start the emergency plan, control the development of the situation, remove, recall, and seal the problem products. At the same time, manufacturers should also explain the truth to consumers and put forward remedial measures, and implement them, to eliminate consumers’ panic psychology and enhance consumer confidence.

Second, the QR code should provide clear and detailed information about food, such as its natural nature, brand, origin, packaging, price, nutrition, ingredients, safety, sustainability, environmental effects, and other information. The information provided by the QR code should be richer and more detailed than what consumers expect, thus reducing uncertainty and strengthening consumers’ buying intentions.

Third, to improve consumers’ awareness of domestic products and make use of consumers’ brand preference, manufacturers should produce products in strict accordance with national regulations to eliminate food safety problems. This is the most fundamental way to solve the problem of food safety, but it requires effective supervision by the government and consumers.

Fourth, it is worth noting that the only unsupported hypothesis refers to the fact that the effect of perceived risk on RQ is not significant. We should further investigate some uncertainty factors that may be involved in the research model. For example, information timeliness and information privacy issues should be considered in the future.

### Limitations and Future Work

Even if we have made the theoretical and managerial contributions, there are three limitations that warrant further research, but the future study directions remain to be discovered. First, considering the limited sample range, future research could incorporate factors such as the age and experience of food traceability consumers as moderators in the theoretical model and examine whether there are differences between different consumer samples based on these characteristics. Therefore, follow-up study is needed. Second, we only researched the Chinese and South Korean consumers in the field of food safety. To strengthen the systematic nature of this study, we would like to compare the results from different countries. Third, we integrated D&M ISS and TPB into PPM to identify the factors that influence the willingness to identify the factors that influence the willingness to use FTS. Future studies could use UTAUT, UTAUT2, TTF model, etc. to test the role of other factors in influencing FTS users’ willingness to switch. Future SEM studies should test the willingness to switch FTS in the field of food safety from a more comprehensive perspective.

Most of the previous studies focused on the traceability of the food chain to retail outlets, so they did not trace the consumer part of the food chain. The consumer component is also important in terms of food safety, so future traceability should be extended to consumers in more countries. If market forces, consumer demand, and government regulations all converge to push supply chain traceability to a new level, food traceability from farm to table will become a reality.

## Data Availability Statement

The datasets presented in this article are not readily available because “It can only be used when publishing in journals that have the basic or share upon reasonable request data policy.” Requests to access the datasets should be directed to linxin@neepu.edu.cn.

## Ethics Statement

Ethical review and approval was not required for the study on human participants in accordance with the local legislation and institutional requirements. Written informed consent from the participants was not required to participate in this study in accordance with the national legislation and the institutional requirements.

## Author Contributions

XL contributed to research design, empirical analysis, article writing; and conducted the methodology, data analysis, and research design. R-ZW developed the original idea for the study. Both authors read and approved the final manuscript.

## Conflict of Interest

The authors declare that the research was conducted in the absence of any commercial or financial relationships that could be construed as a potential conflict of interest.
